# A new species of the planthopper genus *Conosimus* associated with an endemic shrub in southern Spain

**DOI:** 10.1093/jis/14.1.92

**Published:** 2014-07-17

**Authors:** V. M. Gnezdilov, D. Aguin Pombo

**Affiliations:** 1 Zoological Institute of the Russian Academy of Sciences, Universitetskaya nab.1, 199034, Saint Petersburg, Russia; 2 University of Madeira, Campus da Penteada, 9000-390 Funchal, Madeira, Portugal; 3 CIBIO, Centro de Investigação em Biodiversidade e Recursos Genéticos, Universidade do Porto, 4485-601 Vairao, Portugal

**Keywords:** Auchenorrhyncha, Issidae, Issini

## Abstract

The poorly-known genus *Conosimus*Mulsant et Rey, 1855 (Hemiptera: Fulgoroidea: Issidae) includes six species and is briefly reviewed. Adults and fifth instars of a new species, *Conosimus baenai***n. sp.** , are described and compared with other species in the genus. The new species is associated with an endemic shrub, *Echinospartum boissieri* , in Jaen, Spain, in the south of the Iberian Peninsula, one of the richest botanical areas of the Mediterranean Basin.

## Introduction

Issidae is a family of phytophagous Hemiptera distributed worldwide and comprises ≈1,000 species in >150 genera ( Gnezdilov 2010a , 2013a). In the Mediterranean Basin, issids are mainly associated with arid and semiarid landscapes. Knowledge of the issid fauna of the Iberian Peninsula is scarce despite its great diversity and high number of endemic species; about 60 species are known from this area (VMG, unpublished data). The high species richness of the Iberian fauna is likely due to its environmental heterogeneity, which is determined by diverse climatic and geological conditions. Despite the importance of issids for biological conservation as indicators of special biotopes ( Gnezdilov 2007 ) and evolutionary studies with faunogenetic reconstructions ( Gnezdilov 2013b ), the information on these planthoppers is still insufficient.

Among the Western Palaearctic issids, *Conosimus*Mulsant et Rey, 1855 is one of the poorly known genera; its systematics and ecology are not well known. It was established as a monotypical genus for *Conosimus coelatus*Mulsant et Rey, 1855 described from Provence in France ( Mulsant and Rey 1855 ). About 100 years later, Soós (1976) erected the genus *Sphenidius* for *Sphenidius horvathi* described from Valencia in Spain, which was later synonymized with *Conosimus* by Dlabola (1987). Currently, *Conosimus* includes five species known only from the Western Mediterranean Basin: *C coelatus*Mulsant et Rey, 1855*, C horvathi* ( Soós, 1976 ), *C malfanus*Dlabola, 1987*, C noualhieri* Puton, 1898 *,* and *C violantis*Ferrari, 1884 ( Gnezdilov et al. 2014 ) *.*

*C coelatus* is distributed from France, including Corse, via Spain to the Balearic Islands ( Mulsant and Rey 1855 . Dlabola 1987 , Gnezdilov 2010b ). This species was reported from Corse as *C corsicus* as described by Lethierry (1876), but it was placed in synonymy under *C coelatus* by Melichar (1906) . There is also an unconfirmed record of *C coelatus* from Sardinia ( Servadei 1952 ); an additional record of the species from Greece ( Linnavuori 1965 ) is apparently mistaken because of misidentification. Another species of this genus, *C noualhieri,* is known only from Algeria and Morocco (Puton 1898, Gnezdilov 2011 ). *C horvathi* is probably a junior synonym of *C noualhieri* according to its habitus and especially the shape of metope and coryphe. The remaining two species, *C malfanus* and *C violantis,* both known only from the original descriptions and the type specimens, described from two small volcanic Mediterranean islands, referred respectively by Dlabola (1987) as Malfa Island (Italy) and by Ferrari (1884) as Galita Island (Tunisia). In this work, one more species of the genus, *C baenai***n. sp.** , is described from Andalusia (southern Spain), and additional comments on the genus are given.

## Materials and Methods

Sampling was performed by placing a sweeping net below plants of *Echinospartum boissieri* (Spach) Rothm. (Fabales: Fabaceae), beating the plant with a stick, and actively collecting insects from the net with an aspirator. All samples were collected at Sierra del Ahillo (Alcaudete-Jaén) on 14-VII-91, 30-VII-95, and 30-VI-2012 at an altitude of 925 m. Dissected genital segments of the male specimen were boiled for a few minutes in 10% KOH solution. Photos and measurements were taken with a Leica MZ8 stereoscope ( www.leica-microsystems.com ) attached to a JVC KY F7OB video camera ( www.jvc.com ). Images were produced using the software Synoptics Automontage (Syncroscopy, www.syncroscopy.com ) and Adobe Photoshop ( www.adobe.com ). Drawings of the head and genital structures were made with a camera lucida attached to the microscopes Leica MZ95 and Mikmed-1. Terminology of head and larval pits follows Emeljanov (1995 , 2001), except the hypocostal plate, a basal extension of the forewing below and perpendicular to the costal margin, which follows Emeljanov (1971) .

Examined material is deposited in the following collections:

M. Baena collection, Córdoba, I.E.S. Trassierra, Spain

MNCN: Museo Nacional de Ciencias Naturales, Madrid

MMF: Museu Municipal do Funchal (História Natural), Funchal, Madeira, Portugal

MNHN: Muséum national d ` Histoire naturelle, Paris

UMa: Universidade da Madeira, Portugal

ZIN: Zoological Institute, Russian Academy of Sciences, Saint Petersburg, Russia

### Nomenclature

This publication and the nomenclature it contains have been registered in ZooBank. The LSID number is:

urn:lsid:zoobank.org :pub:5D1C6804-85AD-4787-A539-C9267D61DE8C

### Description

*Conosimus baenai* **n. sp.**

Fig. 5 -7, 12-21

**Figures 1-11:**
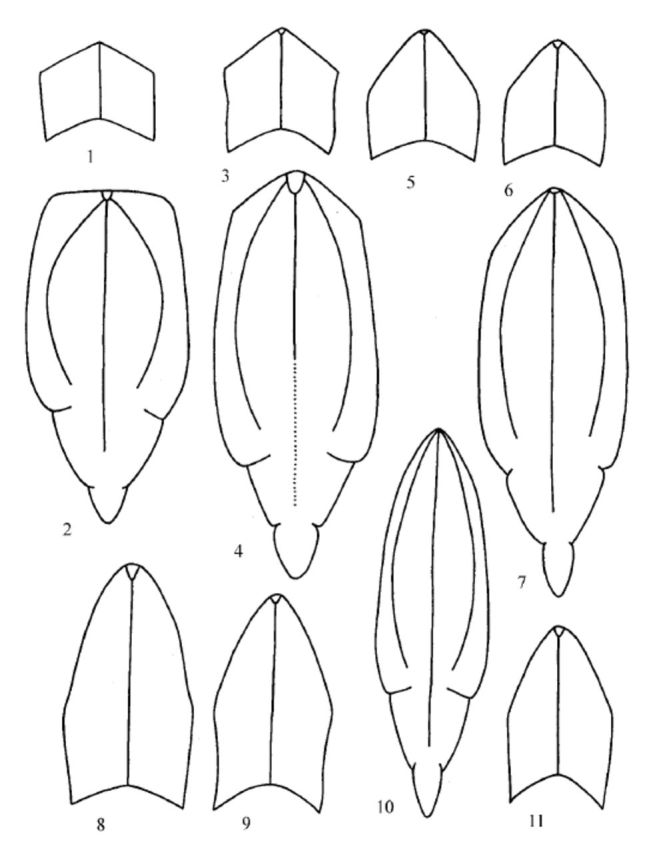
Heads of *Conosimus* species. (1, 2) *Conosimus coelatus* Mulsant et Ray, male (France, MNHN): (1) coryphe, dorsal view; (2) metope and clypeus, frontal view. (3, 4) *Conosimus malfanus* Dlabola, male (holotype, MNHN): (3) coryphe, dorsal view; (4) metope and clypeus, frontal view. (5-7) *Conosimus baenai***n. sp** .: (5) female (paratype), coryphe, dorsal view; (6) male (paratype), coryphe, dorsal view; (7) male (paratype), metope and clypeus, frontal view. (8-11), *Conosimus noualhieri* Puton: (8) female (Morocco, MNHN), coryphe, dorsal view; (9) female (Algeria, MNHN), coryphe, dorsal view; (10) male (syntype, MNHN), metope and clypeus, frontal view; (11) male (syntype, MNHN), coryphe, dorsal view. High quality figures are available online.

**Adult male and female** ( Fig. 5 -7, 12-14, 16-21)

**Description.** Metope long, weakly enlarged below the eyes, with relief median and sub-lateral carinae joint at its upper margin ( Fig. 7 , 14). Sublateral carinae do not reach metopo-clypeal suture. Median carina running up postclypeus. Ocelli absent. Pedicel almost sphaerical. Coryphe 1.5x as long as wide at median line, with relief median carina. Anterior margin of coryphe acutely angulate. Pronotum almost as long as mesonotum at median line, with relief median carina. Pronotum with wide paradiscal fields, with weak carina between the fields and paranotal lobes. Mesonotum with median and lateral carinae. Forewings elongate, narrowing apically, with relatively wide hypocostal plate; radius and median each with two branches, cubitus anterior simple (R 2 M 2 CuA 1), with transverse veins in the apical part of the wing; median furcates after radius in the first third of the wing. Clavus 0.5x as long as the whole wing. Hindwings rudimentary. Hind tibia with 2 lateral teeth distally. First metatarsomere with entire row of 5 intermediate spines and 2 latero-apical spines.

**Figure 12-15:**
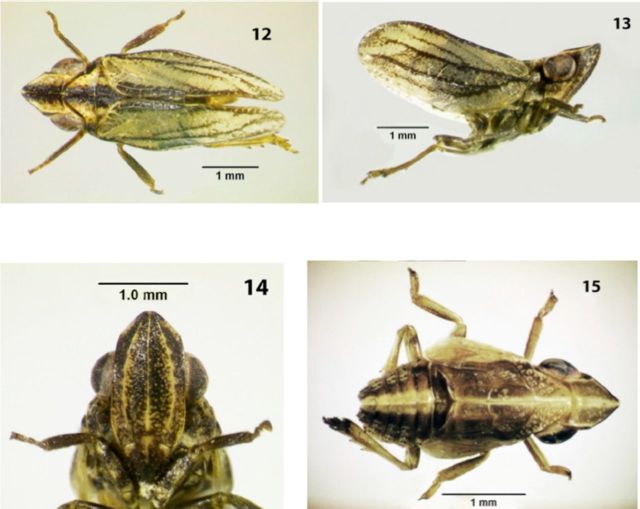
*Conosimus baenai* n. sp.,. (12) female (paratype), dorsal view; (13) same, lateral view; (14) same, frontal view; (15) fifth instar (paratype), dorsal view. High quality figures are available online.

**Coloration.** General coloration light yellow, sometimes greenish-yellow ( Fig. 12 , 13). Metope with dense dark brown or black dots on upper part ( Fig. 14 ). Frontal part of postclypeus or sometimes almost whole postclypeus brown or dark brown. Pedicel brown or dark brown. Coryphe, pro-, and mesonotum with wide median black stripe running up the claval margin of forewings. Paradiscal fields of pronotum each with dark brown or black latero-marginal spot and paranotal lobes with dense dark brown or black dots. Radius and median of forewings framed by dark brown or black stripes. Third segment of rostrum dark brown or black. Fore- and middle femora and tibiae brownish-yellow. Hind femora brown internally. Apices of spines black. Abdominal tergites dark brown or black. Abdominal sternites light yellow, with dark brown dots around setal bases. Male pygofer light yellow, with dark brown or black upper angles. Styles light yellow. Male anal tube light yellow, sometimes, with brown apical margin. Female anal tube dark brown or black apically. Gonoplacs dark brown or black.

**Figure 16-21.**
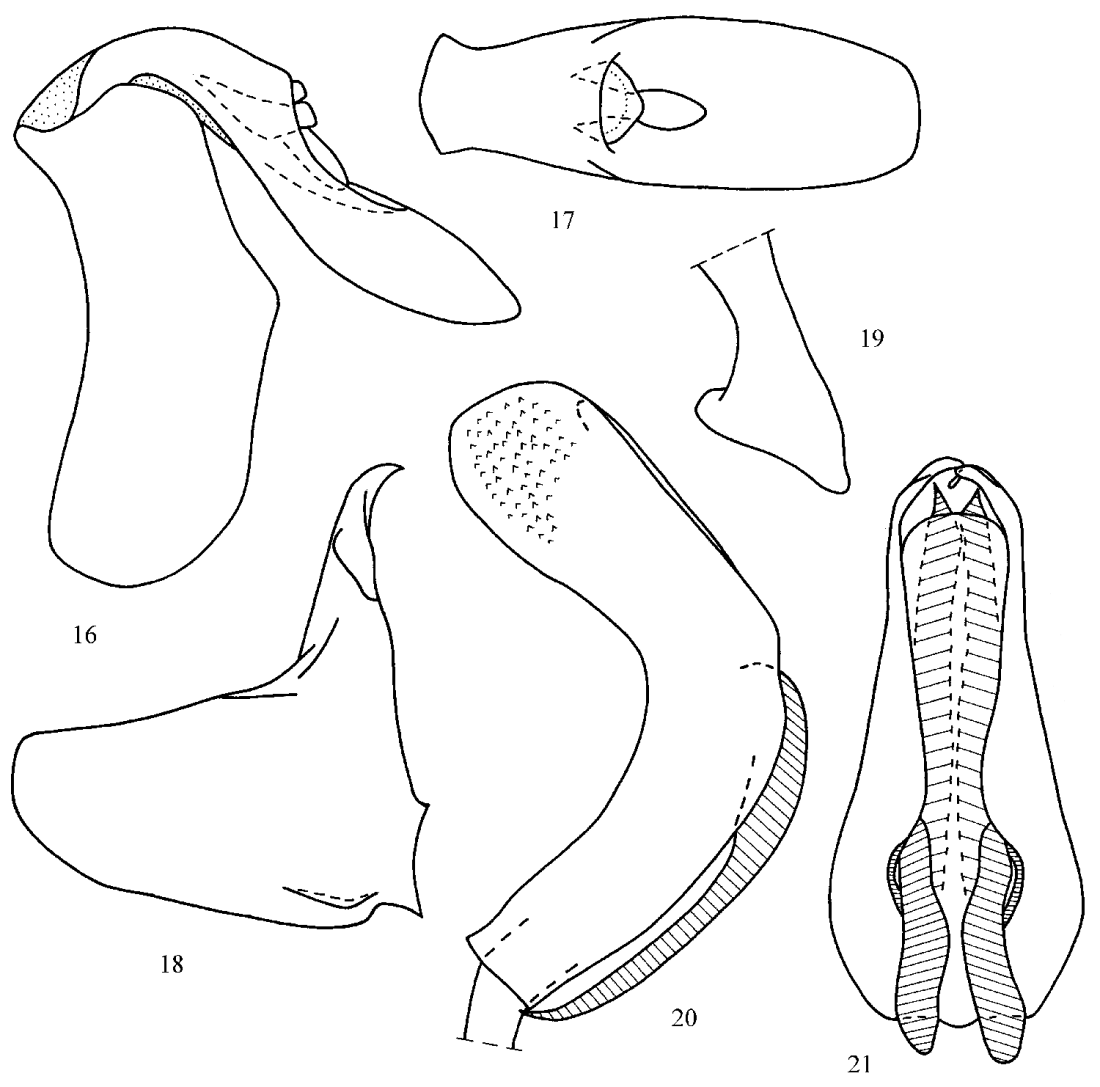
*Conosimus baenai* **n. sp.** , holotype, male genitalia. (16) pygofer and anal tube, lateral view; (17) anal tube, dorsal view; (18) style, lateral view; (19) capitulum of style, dorsal view; (20) penis, lateral view; (21) same, ventral view. High quality figures are available online.

**Male genitalia** ( Fig. 16 -21). Pygofer with hind margin convex medially (in lateral view) and quadrate upper angles ( Fig. 16 ). Anal tube long and narrow, with widely rounded apex (in dorsal view) ( Fig. 17 ). Anal column short. Phallobase without processes, curved at obtuse angle, rounded apically (in lateral view), with long, widely rounded ventral lobe ( Fig. 20 , 21). Ventral margins of the phallobase turned out under the aedeagal hooks ( Fig. 21 ). Aedeagus with pointed apical processes and a pair of long narrowing apically ventral hooks. Style with straight hind margin and rounded caudo-dorsal angle ( Fig. 18 ). Capitulum of style narrowing apically (in dorsal view) ( Fig. 19 ), on long neck, lateral tooth wide.

**Female genitalia.** Hind margin of sternum VII concave medially. Anal tube long and narrow. Anal column short. Gonoplacs with no keels.

**Total body length.** Males: 4.1-4.4 mm. Females: 4.7-5.0 mm.

**Fifth instars** ( Fig. 15 ).

**Description.** Metope with median carina reaching basal part of postclypeus and with sublateral carinae not reaching the metop-oclypeal suture. Median and sublateral carinae j oint at upper margin of metope forming acute angle. Metope with 22-24 sensory pits in two rows between lateral keels and sublateral carinae at each side. Coryphe with no median carina, anterior margin acutely angulate, lateral margins parallel to each to other, posterior margin concave. Rostrum reaching hind coxae. Pronotum with tiny median carina; discal+posterolateral group of pits with 12 pits and paradiscal group with 2 + 4 pits on each side. Mesonotum with tiny median carina and relief lateral carinae; median paradiscal group of pits with 5 pits on each side. Metanotum with tiny median carina and smooth lateral carinae, every median paradiscal group with a single pit. Forewing pads reaching middle of abdominal tergite IV. Each forewing pad with 2 sensory pits on a longitudinal row and 2 isolated pits on the sides. Tergite IV with 4 lateral pits; tergite V with 6 pits; tergites VI‒VIII with 5 lateral pits; tergite IX with 3 lateral pits (the numbers are indicated for each side). Hind tibia with 2 lateral teeth distally and 8‒9 apical spines. First metatarsomere with 7‒8 apical spines.

**Coloration.** Metope light yellow except brown or dark brown apical angles between median and sublateral carinae. Clypeus light yellow. Coryphe, pronotum, except light yellow distally paradiscal fields, meso- and metanotum, and forewing pads basally brown, dark brown, or black. Median line of the head and body with light narrow stripe on pro-, meso-, and metanotum and wide on the coryphe and abdomen. Forewing pads light yellow except basal parts. Pedicel, hindwing pads, and paranotal lobes brown, dark brown, or black. Abdominal tergites brown, dark brown, or black with light yellow patches. Abdominal sternites and legs brownish yellow. Apices of spines black.

**Total body length.** 2.8–3.3 mm.

### Type material

HOLOTYPE: ♂, Sierra del Ahillo, Alcaudete-Jaén, Spain, 37° 36.692' N, 4° 1.782' W, [on] *Echinospartum boissieri* , 30-VII-1995, M. Baena leg. (MNCN).

PARATYPES: 4 ♂♂ *,* Sierra del Ahillo, Al-caudete-Jaén, 37° 36.692' N, 4° 1.782' W, [on] *Echinospartum boissieri,* 30-VII-1995, M. Baena leg. (MNCN - 2 ♂ *;* M. Baena - 1 ♂; ZIN - 1 ♂ *)* ; 1 ♀, 2 nymphs (fifth instar), same locality, 14-VII-1991, M. Baena leg. (ZIN); 8 ♂♂ *,* 7 ♀♀, 5 nymphs (fifth instar), same locality, 30-VII-2012, M. Baena leg (ZIN - 2 ♂♂, 2 ♀♀, 2 nymphs (fifth instar; MNCN - 1 ♂ *,* 1 ♀, 1 nymph (fifth instar); MNHN - 1 ♂, 1 ♀; MMF - 1♂, 1 ♀, 1 nymph (fifth instar); UMa - 1 ♂, 1 ♀, 1 nymph (fifth instar); M. Baena - 2 ♂♂, 1 ♀).

**Etymology.** The new species is named *baenai* as a masculine Latinized noun derived from the surname of Manuel Baena, a Spanish he-mipterologist, in recognition for his kind support to this study and his contribution to the taxonomy of Iberian Hemiptera.

**Diagnosis.** The new species clearly differs from other species of the genus in the coloration of forewings and veins. Although all species of the genus have veins the same color as the wings (not marked), usually light brown or light brown yellowish, *C. baenai***n. sp.** has light yellow, sometimes greenish yellow wings in contrast to radial and median veins framed by dark brown or black stripes ( Fig. 13 ).

**Habitat.** The new species was found in a habitat dominated by thorny shrubs of *Echinospartum boissieri* (Spach) Rothm. (Fabaceae) in a medium-high mountain area ( Fig. 22 and 23). This rare shrub is endemic to the Baetic Range in southern Spain and conforms scrublands on calcareous substrate ( Valdés et al. 1987 ), being found among the natural communities *of Pinus halepensis* ( Torres et al. 1999 ). The bloom time and fruits of this yellow-flowering shrub occurs from July to August.

**Fig 4.**
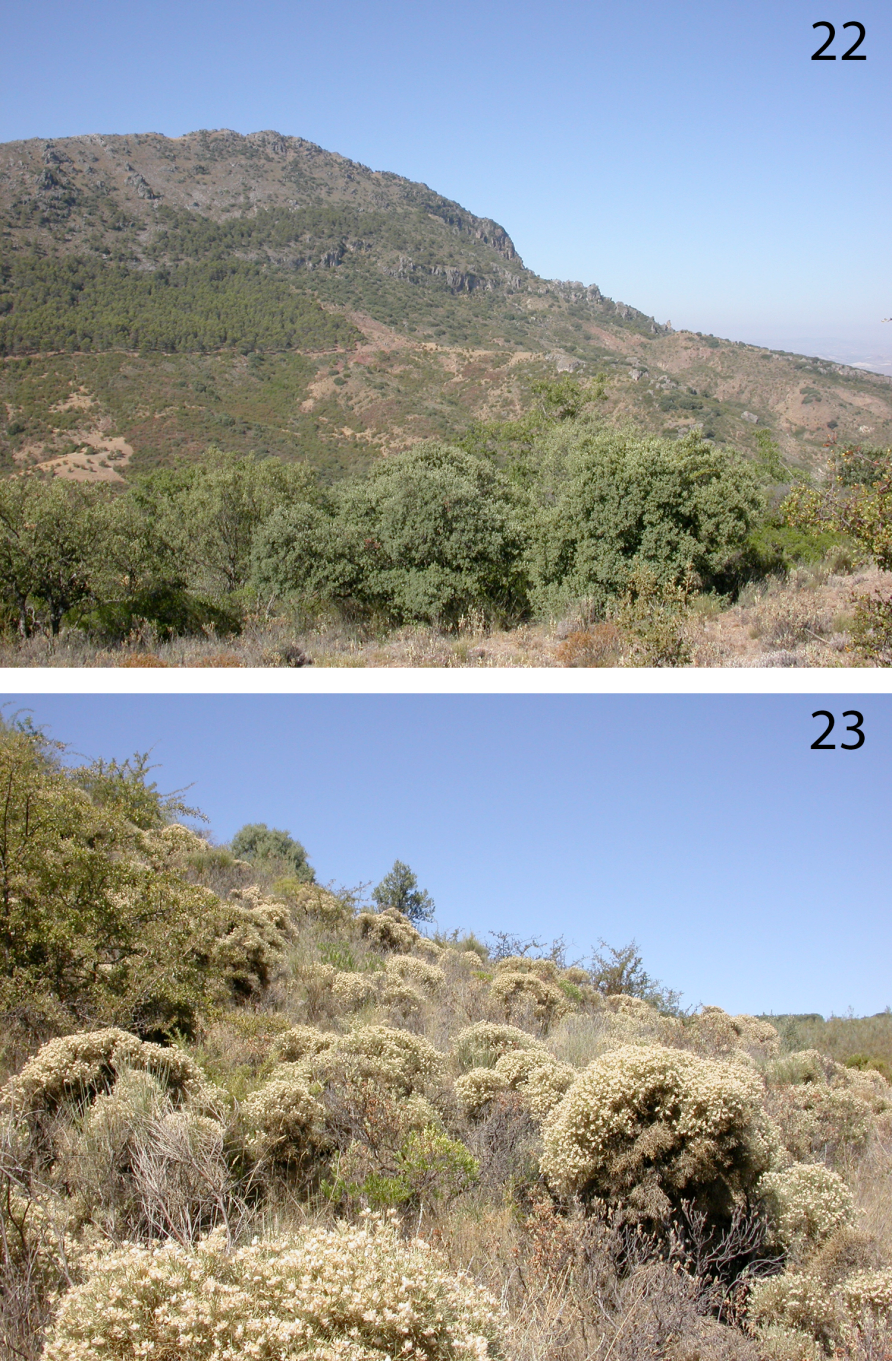
Type locality of *Conosimus baenai* n. sp., Spain, Sierra del Ahillo, Alcaudete-Jaén. (22) landscape photograph. (23) bushes of *Echinospartum boissieri* , the planthopper host plant at site. High quality figures are available online.

**Distribution and biology.** The species is known only from the type locality, Sierra del Ahillo, near Alcaudete in Jaen. It was collected on *E. boissieri* when the plant was in fructification at 925 m above sea level, but the plant grows at higher altitudes. Nymphs and adults occur in July on *E. boissieri* .

## Discussion

According to the length of coryphe medially, the five species of *Conosimus* described so far were recognized in two groups: species with short coryphe ( *C. coelatus* , *C. malfanus* , *C. violantis* ; Fig. 1 , 3) and species with long coryphe ( *C. noualhieri* , *C. horvathi* ; Fig. 8 , 9, 11). In both groups, the females have coryphe longer than males have. Since the width of the metope is correlated with the length of the coryphe, species with long coryphe have narrower metope and species with short coryphe have wider metope ( Fig. 1 –11). *Conosimus baenai***n. sp.** occupies intermediate position between these two groups according to the length of coryphe ( Fig. 5 ‒7). According to Dlabola’s (1987) illustrations, species of the genus are slightly different from each other in the structure of male genitalia. Further study is in need to determine whether there are genital differences that can be useful for the identification of species.

Three species of *Conosimus* occurring on the Iberian Peninsula seem to differ in distribution: *C. horvathi* is only known from Valencia (Eastern Spain), whereas *C. baenai***n. sp.** and C *. coelatus* are reported from Andalusia (Southern Spain), Jaen, and Granada ( Dlabola 1987 ), respectively. The latter also occurs in the Balearic Islands (Dlabola 1985, Gnezdilov 2010b ).

The ecology of *Conosimus* is almost unknown. Published information for *C. malfanus* indicates that this species is associated with halophile vegetation in its type locality ( Dlabola 1987 ). However, *C. coelatus* was collected recently by the first author in Provence (Vaison-la-Romaine) on garriga vegetation (VMG, unpublished data), and *C. baenai***n. sp.** was found associated with scrublands of *E. boissieri* in Andalucia (Sierra del Ahilho). These data suggest that these species are ecologically diverse, occupying different types of habitats that range from sea level to middle high mountain altitudes. The association of *C. baenai***n. sp.** with a rare shrub, endemic to southern and southeastern Spain ( Valdés et al. 1987 ), suggests that it may follow the distribution of its host plant along the Baetic System. This system of multiple mountain ranges is aligned in a southwest‒northeast direction from western Andalusia to Murcia and Valencia; it is extremely rich and outstanding in endemic plants (418 taxa belonging to 43 families) (Pé-rez-García et al. 2012). The association of *C. baenai* with *E. boissieri* is particular interesting because *Echinospartum* is endemic to the Iberian Peninsula and France. Because of its geographical isolation, this plant genus diversified into four endemic species representing two ecologically (sidicicolous vs. calcicolous) unrelated groups ( Talavera 1999 ). Because the species of *Echinospartum* differ in flavonoid composition, plant secondary metabolites (Bermejo et al. 1987), known to be insect-feeding deterrents, a degree of host plant specialization is likely to occur in *C. baenai* . Further analysis of the fauna of *Conosimus* associated with *Echinospartum* spp. not only could give ideas about the diversification of the *Conosimus* species but also may contribute in defining the areas of *Echinospartum* , which may be recognized as different chorological units ( Rivas Martinez 1975 ) and prioritized for conservation purposes.
